# The Commission for the Study of Tuberculosis

**Published:** 1904-09

**Authors:** 


### THE COMMISSION FOR THE STUDY OF TUBERCULOSIS.

A BILL was passed at the last session of the Legislature creating a commission for the study and investigation of tuberculosis in the State of Georgia. The commission will be composed of one physician from each Congressional district and ten from the State at large. These commissioners will undertake to investigate the status of the disease in the State with a view to suggesting such measures as will tend to check the spread of it and to throw around the public such safeguards as science has shown are effective. The members of the commission are to be appointed by the Governor. Their names have not yet been announced.

It is a very hopeful sign when the State wakes up to a tardy recognition of the trite formula *salus populi suprema lex est,* and with its relentless pursuit of the boll weevil will turn aside to attack the menace that is of larger importance than *lese-majesté* to King Cotton, that stalks through the land.

The inspiration of this movement sprang from Dr. George Brown, Secretary of the Tuberculosis Congress, and the passage of the bill was due to the enlightened efforts of the Fulton county representatives and Senator Jordan.

